# Characterization of the weathered basement rocks in the Dongping field from the Qaidam Basin, Western China: significance as gas reservoirs

**DOI:** 10.1038/s41598-020-73898-0

**Published:** 2020-10-07

**Authors:** Wei Yang, Jiangong Wang, Feng Ma, Yongshu Zhang, Yadong Bai, Xiujian Sun, Hongzhe Li, Jing Zhang, Pu Wang

**Affiliations:** 1 Research Institute of Petroleum Exploration and Development-Northwest (NWGI), PetroChina, Lanzhou, 730020 China; 2 Research Institute of Exploration and Development, PetroChina Qinghai Oilfield Company, Dunhuang, 736202 China; 3 Key Laboratory of Reservoir Description, CNPC, Lanzhou, 730020 China

**Keywords:** Natural gas, Geology

## Abstract

Reservoir quality is a critical risk factor in basement reservoirs. Researches into basement reservoirs by petrographic analysis combined with X-ray diffraction, log identification, electron microscopy, field emission scanning electron microscopy, porosity and pulse-decay permeability and core analysis have provided insights into the characterization of the commonality, diversity and difference of the weathered basement rocks as gas reservoirs in the Dongping field. Geological structures, lithology and near-surface processes control the reservoir physical property together. From Wellblock Dp 3 to Wellblock Dp 17, the high uplift gradually transforms into the low slope area towards the center of basin, with the lithology changing as well, which results in different degrees of fracture development in the bedrock in different wellblocks. The basement lithologies are granite, granitic gneiss, and limestone with slate in Wellblock Dp3, Dp1 and Dp17, respectively. Though they all provide effective reservoir space for gas accumulation, the productivity of nature gas shows significant differences. Fractures are the main store space in the three wellblocks. The development of fractures gives rise to secondary porosity around them because of physical weathering and chemical dissolution, but they generate many inhomogeneous fractures and secondary solution pores, whether on the planar distribution or in vertical. In Wellblock Dp3, high angle fractures were generated under the action of structural stress mechanism, with a large number of secondary pores. The porosity is between 0.1 and 23.2%. In Wellblock Dp 1, low angle fractures were the main storage space, with plenty of solution pores mainly in melanocratic minerals. The porosity is between 0.1 and 18.8%. In Wellblock Dp 17, where short and dense fractures developed, the porosity is between 0.1 and 10.3%. The data indicate that the granite in the uplift in Wellblock Dp3 has better reservoir properties due to the stronger physical weathering and chemical dissolution. As the porosity gradually decreases towards the slope and low-lying area, the more favorable exploration area should be the uplift and slope area in the depression area. However, the effective caprock developed locally in Wellblock Dp3, which affected the gas accumulation. Meanwhile, the reservoirs’ petrophysical properties showed distintive variation with different depths in different wellblocks. High productivity layers are under the 200 m, 100 m and 200 m depths from the top of the basement rocks in Wellblock Dp 3, Wellblock Dp 1 and Wellblock Dp 17, respectively. This suggestion in this study will be of significance for guiding oil and gas exploration in front of the Altun Mountains.

## Introduction

With the increasing demand for energy, the basement reservoir as a special type of hydrocarbon reservoir has been an important target for exploration all over the world^1,2^. Mostly found on platforms or in intermontane basins^3,4^, they are typically featured by very complex, hard and solid basement rocks that are metamorphic and igneous. However, a large number of inhomogeneous fractures and secondary dissolution pores^5–7^ may occur due to weathering, leaching, crumbling, decomposition, dissolution, erosion and strong tectonic movement caused by long-term uplift and fold formation, all resulting in the change of reservoir space. With the discovery of basement reservoirs in the Jiuquan Basin, the Bohai Bay Basin, the Liaohe Basin, the Qaidam Basin and the Santanghu Basin^8–12^, increasing importance and attention has been attached to the exploration of oil and gas reservoirs in the basement rocks of China.

In recent years, the exploration of basement hydrocarbon reservoirs in Qaidam Basin has continuously exhibited substantial progress with the discovery of basement reservoirs in the Dongping area in front of Altun mountain. Well Dp1, aiming at basement rock reservoir, was drilled in the Dongping area, with daily production of 11.2 × 10^4^ m^3^, and subsequent drilling confirmed that the hydrocarbon reservoir as the largest continental basement rock gas reservoir in China^12^. Daily production of 3.6 × 10^4^ m^3^ was obtained in the basement rocks in well Dp 3 , and multilayered gas reservoirs have also been discovered in the Paleogene stratum. Well Dp 17 has secured 5.0 × 10^4^ m^3^ daily in the basement rocks , with the forecast reserves of natural gas reachingup to 2.280 × 10^8^ m^3^. Previous studies suggest that the gas reservoir is a large and blocky basement-rock reservoir that is featured by a unified gas and water interface and a high gas column^12–14^. Deeper basement zones dominated by fractured and cavernous-fractured reservoirs often have deceptively high initial production, leading to the overbuilding of production facilities. Therefore, when the field is placed on production, rapid production declines may be experienced since the reservoirs only have fracture porosity and minimal matrix porosity. Fracture reservoirs with much heterogeneity is typical in basement rocks^15,16^. The main purpose of this study is to investigate the characteristics of the basement rock reservoir and the effect of gas reserve tests, in addition to the distribution of favorable prospecting layers for the future exploration in the Qaidam Basin.

## Geological setting

**Figure 1:**
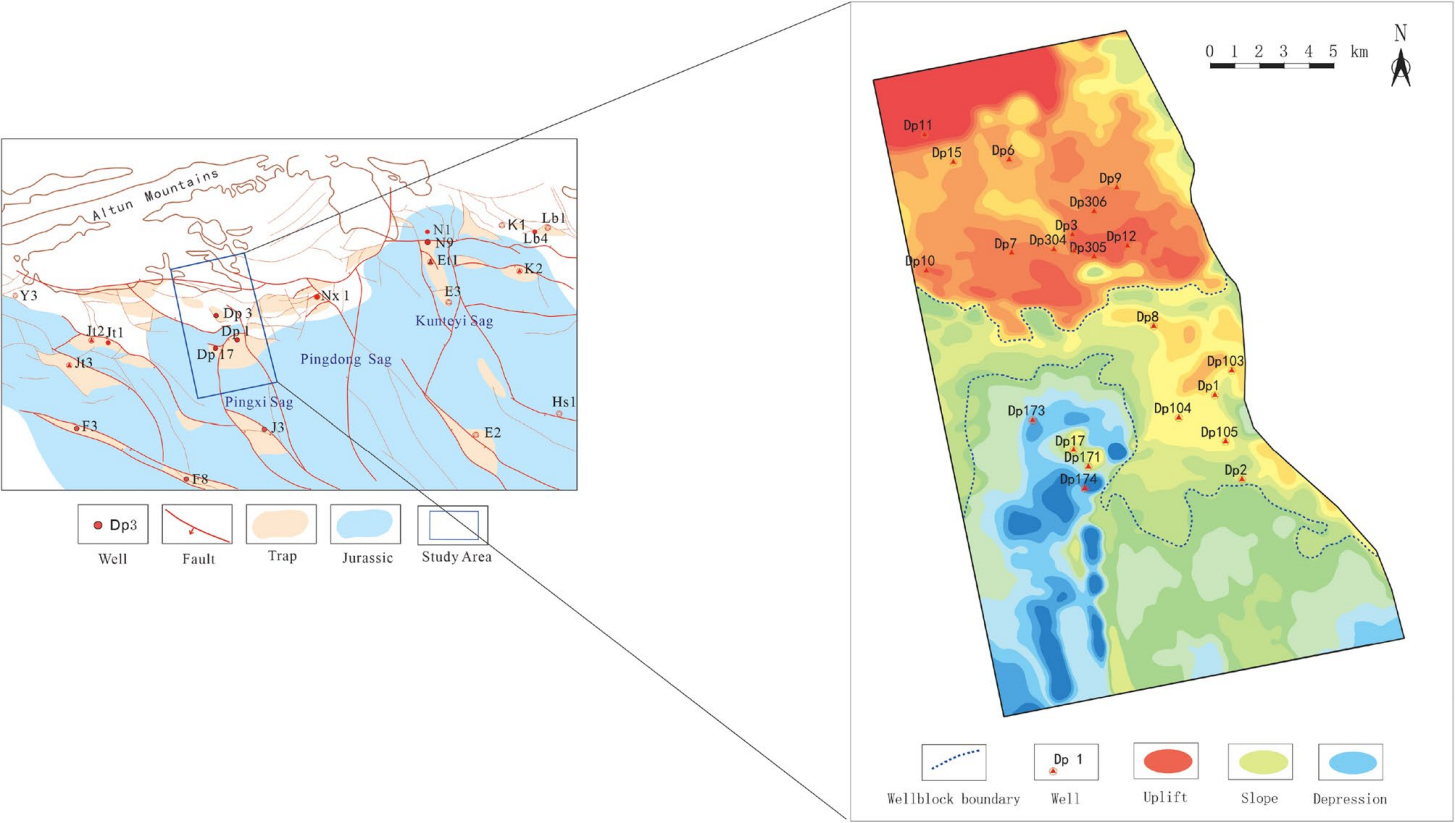
Partitioning of the three wellblocks in the Dongping area. The regional structural map and the paleogeomorphogloic map were compiled based on the fine interpretation of horizons and faults with seismic data in the Dongping area, and then with Geomap 3.6 version mapping software.

Located east of the Altun Mountains in the north of the Qaidam Basin, the Dongping field covers an area of approximately 6726.9 km^2^ ,with Jianding Mountain to the west to Kunteyi to the east, and Jianshan to the south, where the basement is a composite one composed of Paleozoic metamorphic rocks and multistage granites. Controlled by north–south striking faults, it is marked by a paleo-uplift dipping south. During drilling in the Dongping gas field, exploration breakthroughs were made in Wellblock Dp3, Dp1 and Dp17 (Fig. 1). Wellblock Dp3 is located in the high fault-uplift zone in front of the Altun Mountains and Well Dp 3 was drilled at a depth of 1850 m^17^. Wellblock Dp1 is situated on the slope in the eastern part of the nose structure, and the basement rock was drilled at a depth of 3072 m in Well Dp1, while Wellblock Dp17 is located in the southwestern part of the nose structure which is at a lower structural position towards the basin. The basement rock was drilled at a deeper depth of 4299 m in the well Dp17.

The main deposits in front of the Altun Mountains are the Jurassic, Paleogene and Neogene strata. The strata uplifted influenced by tectonic movements in front of the Altun Mountains, and the Jurassic strata, a huge natural gas kitchen, turns thinner as it approaches the mountains. However, the huge thick mudstone deposited in the Jurassic strata are mainly Type II_2_-III with high content of organic matter of 1.5–3.5% and the vitrinite reflectance is more than 2.0%^12,13^, from where the generated gas was mainly transported by the deep fractures connecting the Jurassic source rock with the basement rocks in front of the Altun Mountains. Meanwhile, the Lulehe Formation in front of the Altun Mountains contains abundant anhydrite that was deposited as cement and filled the fractures and pores, leading to decreased porosity and to the formation of a tight caprock for hydrocarbon accumulation in the three wellblocks^15^. However, the anhydrite-bearing caprock is only fine local caprock that becomes thinner from the lowland to the uplift and provides super sealing conditions for the gas reservoir in Wellblock Dp1 and Wellblock Dp17, whereas it is missing in Wellblock Dp3^15^. Jurassic gas source and anhydrite-bearing caprock are advantageous conditions for the formation of the Dongping field,but the difference of gas yield is decided by basement rock reservoir.

## Sample collection and test

Numerous basement samples were collected from the well cores of the three wellblocks in the Dongping field and analyzed^15^.

The micro-morphology, texture and fabric were examined by visual observation of the cores, and then by thin section observation with regular and casting thin sections, field emission scanning electron microscopy image. Approximately 300 points were counted in the thin sections by using the point counting method of Gazzi-Dickinson. This method determined the topographic characteristics of the fractures and pores^18^.

Mineral compositions were determined by X-ray diffraction where a Rigaku D/maxrA12KW rotating anode X-ray diffractometer with Cu K-alpha radiation (40 kV, 100 m) was employed. After that, stepwise scanning measurements were carried out at the rate of 4/min within the range between 3° and 85° (2θ)^15^. The X-ray diffraction analysis was also combined with thin section observation.

A total of 60 samples without obvious fractures were measured for effective porosity and permeability with a PoroPDP-200 automated permeameter-porosimeter. The samples were made into cylinders 3 cm long and 2.5 cm in diameter. The pulse-decay permeameter-porosimeter can determine porosity and pore volume at overburden pressures up to 70 MPa with helium. The effective porosity values were calculated with Boyle's law and ranged from 0.01 to 40%. The equivalent air permeabilities at a user-specified pressure were also calculated, ranging between 0.001 mD and 10,000 mD^19^_._

These analyses were carried out at the Key Laboratory of Reservoir Description, Northwest Branch of PetroChina Exploration and Development Research Institute (NWGI).

## Results

### Lithology

The basement is characterized predominantly by two types of rocks in the Dongping field: magmatite rocks and metamorphic rocks. Granitic intrusive rocks are the main lithology of the magmatite rock, whilemetamorphic rocks include granitic gneiss, shallow metamorphic limestone and slate.

**Figure 2:**
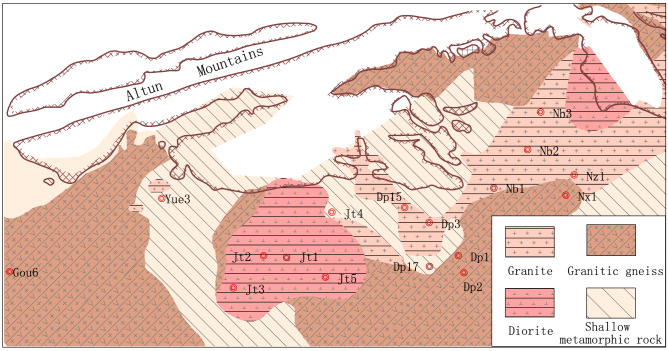
Lithofacies distribution in the front of the Altun Mountains. This map was drafted by using lithologic logging data of the drilled wells and the seismic facies analysis of the seismic data in the Dongping area before finally compiled with the Geomap 3.6 version mapping software.

**Figure 3:**
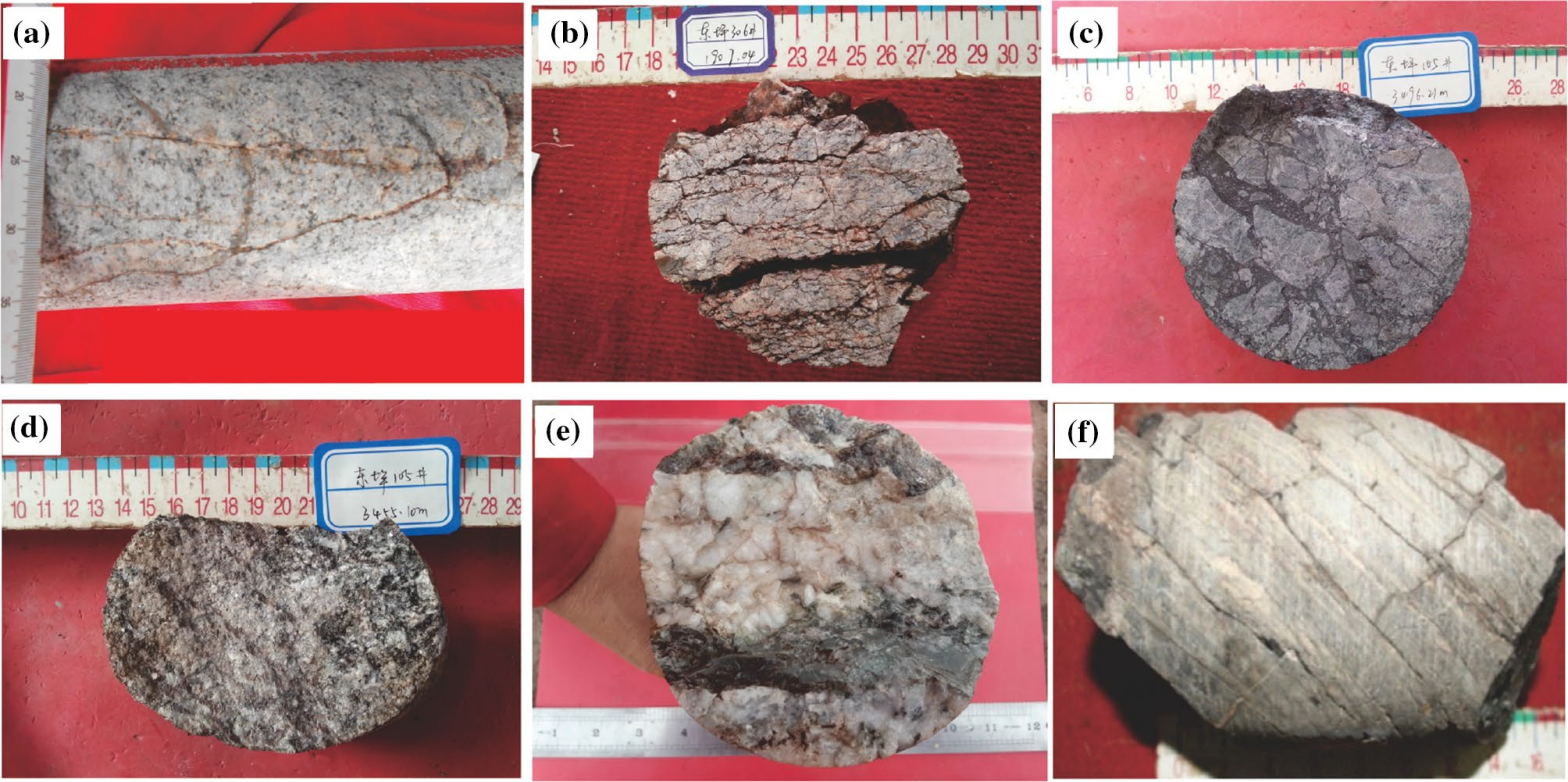
Photographs of cores of basement rocks in different wellblocks in the Dongping area. (**a**) Granite with massive and medium-grained structure in Wellbolck Dp 3; (**b**) broken granite core and fractures developed; (**c**) granitic gneiss; (**d**) granitic gneiss, gneissic structure, with quartz and feldspar forming a light-colored layer and, iron-magnesium minerals forming a dark layer in a sheet-like structure in Wellbolck Dp1; (**e**) shallow metamorphic limestone in conjunction with thin layer slate in Wellbolck Dp 17; (**f**) thin layer slate containing high calcite in well DP 17 in Wellbolck Dp 17.

Constituting the basement of Wellbolck Dp 3 (Fig. 2), granites are massive and medium-grained (Fig. 3a, b). Results of X-ray diffraction (XRD) analysis (Table 1) show that the dominant constituents of granites are quartz and feldspar minerals, whose contents were up to 34.2–46.9% and 17.0–40.0%, respectively, though leucocratic and biotite—and plagioclase-rich varieties have also been identified.

**Table 1:** Mineralogical compositions of the basement rock samples in different wellblocks.

Sample ID	Mineral content/%						
	Quartz	Potassium feldspar	Plagioclase	Total clay	Total carbonate	Biotite	Lithic fragment
Dp3 Wellblock	34.2–46.9	1.0–6.2	16.0–33.8	3.5–11.5	0–12.0	6.0–17.0	0–3.6
Dp1 Wellblock	20.1–62.3	0.6–9.0	2.0–42.9	3.0–32.9	0–11.3	1.1–15.2	0–17.4
Dp17 Wellblock							
Limestone	5.6–13.2	0.1–0.4	0.9–3.7	6.2–15.3	61.1–77.8	–	3.9–7.1
Slate	25.8–31.2	0.2–0.5	1.0–5.1	23.3–36.2	27.4–36.1	–	4.9–13.5

Granitic gneiss form the basement of Wellbolck Dp 1 (Fig. 2). The main minerals are quartz, plagioclase, clay, as well as small amounts of biotite and amphibole, which are medium to coarse structure. It has a distinct gneissic structure, with quartz and feldspar forming a light-colored layer and iron-magnesium minerals forming a dark layer in a sheet-like structure (Fig. 3c,d).

Shallow metamorphic limestone occurred in the basement of Wellbolck Dp 17 and in conjunction with thin layer slate (Fig. 2, 3e,f). High content of calcite can be found in the limestonewhich also contains a small amount of minerals such as quartz, feldspar and clay. The slate strip is mainly composed of clay, calcite and quartz, and contains a small amount of debris such as feldspar.

### Types of reservoir space

**Figure 4:**
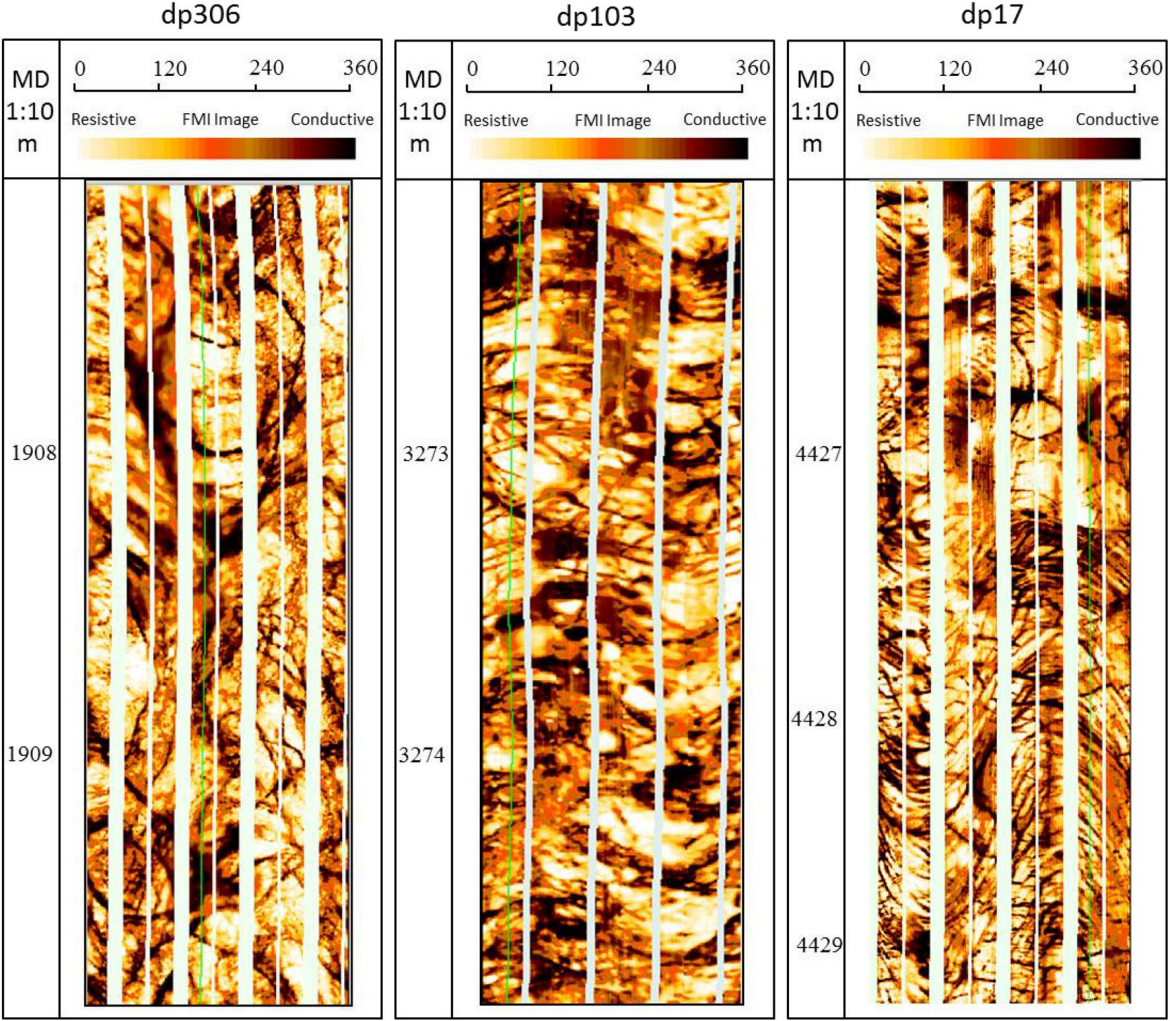
FMI imaging logging characteristics of fractures and dissolution pores in different wellblocks in the Dongping field. (Well Dp306 in Wellblock Dp3, Well Dp103 in Wellblock Dp1, Well Dp17 in Wellblock Dp17).

**Figure 5:**
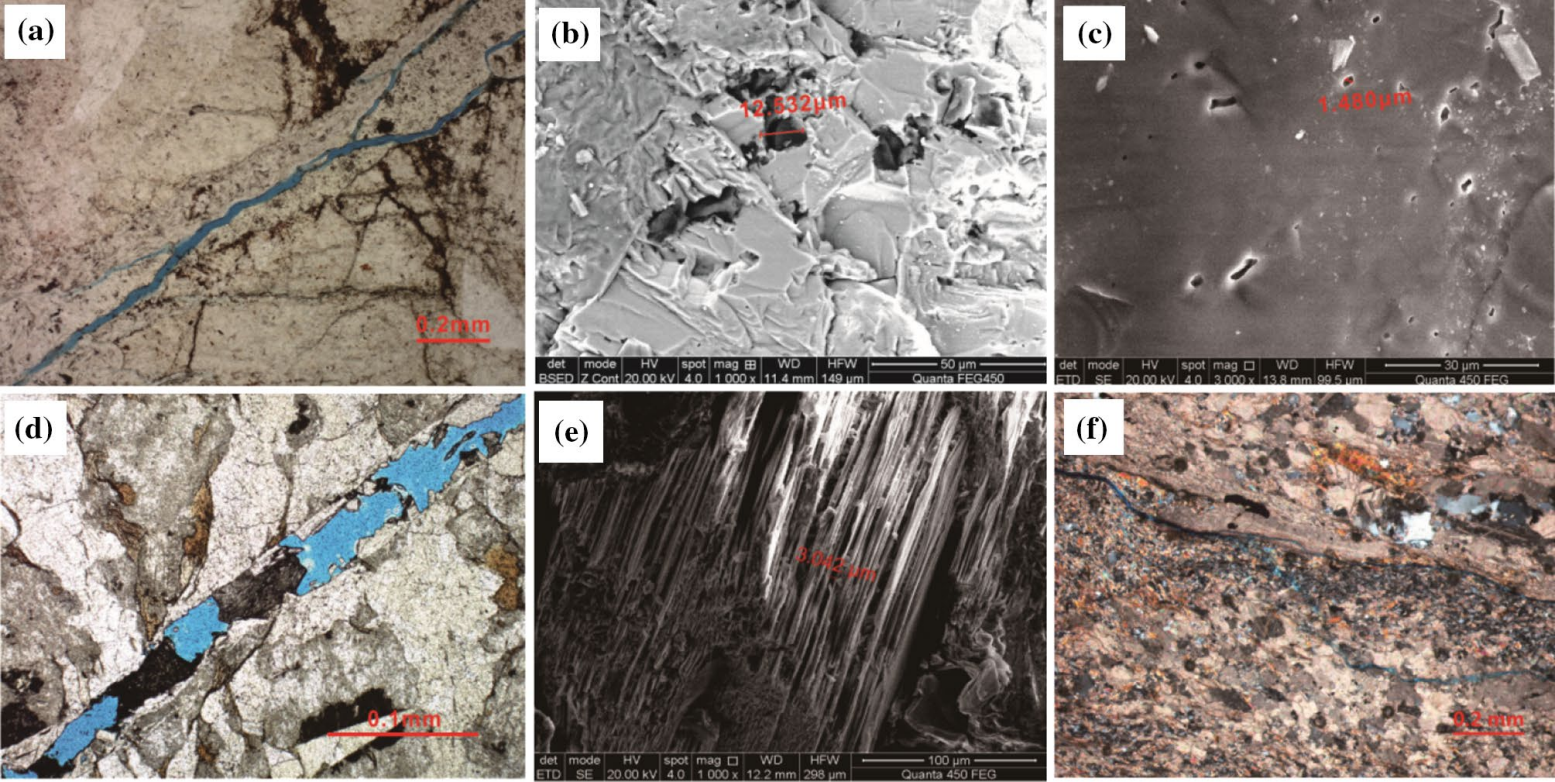
Photographs of thin sections and scanning electron microscope (SEM) images of fractures and pores in basement rocks. (**a**) Fractures in granite in Wellblock Dp3. (**b**) Solution pores developed in groups at the edges of minerals in granite in Wellblock Dp3. (**c**) Solution pores sporadically developed on mineral surfaces in Wellblock Dp3. (**d**) Semi-filled high-conductivity fractures in Wellblock Dp1. (**e**) Intracrystalline pores between mica sheets in granitic gneiss in Wellblock Dp1. (**f**) Fractures and solution pores in limestone in Wellblock Dp17.

Wellblock Dp3 is located in the high fault-uplift zone in the northern part of the nose structure. Due to the high content of brittle minerals of quartz and feldspar in granite, a large number of high angle fractures (45°–75°)are easily generated under the action of structural stress mechanism. The micro-resistivity scanning imaging logging (FMI) data showed that the fractures developed well, exhibiting dark and irregular strips with wide diameters, irregular seam boundaries, relatively large changes in the appearance and significant extension (Fig. 4). In addition, a great many dissolution fractures are easily formed around the wide fractures, with a larger number, a narrower width, and a different degree of extension compared with the structural fractures. The average density of fractures is 1.56 pieces per meter which is identified by core observation and FMI in Wellblock Dp3. The width is generally 0.03–1.346 mm by thin section (Fig. 5a) and individual fractures can be more than 5 mm wide. Dissolved pores are characterized by circle-ish shapes and different sizes. The diameter of the pores observed in the imaging range is several hundred nanometers with the maximum being around 2.5 µm. They existed in two forms: in groups at the edges of minerals that were extensively dissolved, and sporadically developed on mineral surfaces (Fig. 5b,c).

Fractures in Wellblock Dp1 are mainly oblique fractures which were formed by high angle fractures with many low angle fractures(15°–45°) (Fig. 4). In general, the planar heterogeneity of the bedrock lithology will cause the horizontal stress to be heterogeneous in the horizontal direction. The rock's in-situ stress will inevitably grow with fining particle sizes and increasing argillaceous contents^20^. In addition, the difference in rock mechanics caused by the change of lithology will hinder the extension of the fractures and even change their directions^21^. The argillaceous content in granitic gneiss is higher than that in granite, which led to the change of fracture direction. The average density of fractures is 7.65 pieces per meter which is identified by core observation and FMI in Wellblock Dp1. Most fractures are semi-filled high-conductivity fractures that are not completely filled with conductive minerals. They appear as irregular black strips, mostly lenticular, with visible black spots on the edges of the fractures which are formed by the dissolution process (Fig. 4). The width of the fractures observed in the thin sections is concentrated at 0.03–0.575 mm, among which the individual ones are up to 2 mm wide (Fig. 5d). However, a plenty of intercrystalline pores have developed between the mica sheets with diameters of up to 7 μm, existing as an important pore type in granitic gneiss (Fig. 5e).

The main minerals in the bedrock of Wellblock Dp 17 are calcite, andwhere? a large number of cleavage fractures developed (Fig. 4) with anaverage density of 1 piece per meter which is identified by core observation and FMI in Wellblock Dp17. These fractures developed mainly in groups, relatively fine and with similar appearance. Their widths mainly concentrate within the 0.05–0.35 mm range, and the widest can be wider than 2.5 mm . Meanwhile, a large number of dissolved pores and dissolved caves developed on the surface and edges of the calcite due to strong dissolution (Fig. 5f).

### Petrophysical properties of reservoirs

**Figure 6:**
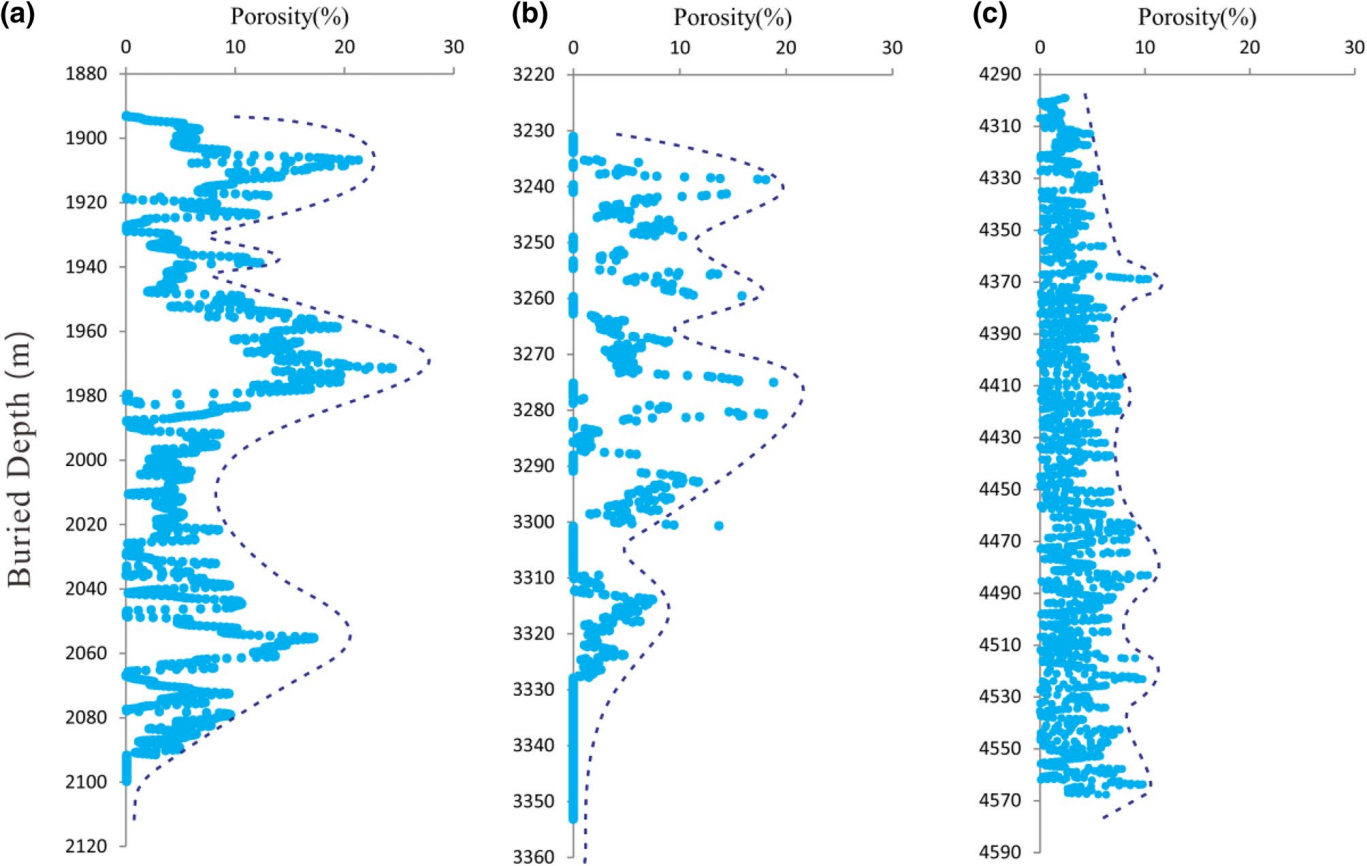
Distribution of porosity with increasing depth in the basement rock reservoirs. (**a**) Well Dp306 in Wellblock Dp3. (**b**) Well Dp103 in Wellblock Dp1. (**c**) Well Dp17 in Wellblock Dp17.

The effective porosity and permeability of some of the samples were measured based on the different lithofacies in the three wellblocks by helium measurements. The results show that in Wellblock Dp3, the maximum effective porosity of granite is 11.0% with an average of 6.1%, and the maximum permeability is less than 1.1 mD. These data show the porosity and permeability developed in the basement rock that did not developed fractures. However, according to acoustic curve calculation, the are between 0.1 and 23.2%, with about 25.6% of them being greater than 10%. Two peak areas appear in the porosity within 100 m from the top of the bedrock, and the third appeared between 2020–2080 m as the burial increased due to the strong physical weathering and heterogeneous chemical dissolution along the fractures (Fig. 6a).

The maximum effective porosity of granitic gneiss in Wellblock Dp1 is 9.6% by helium measurements compared with the average of 6.5%, and the maximum permeability is less than 0.8 mD. However, acoustic curves show that the porosities are between 0.1 and 18.8%, whereas about 9.7% of the porosities are greater than 10% (Fig. 6b). Three consecutive peak areas appear in the porosity within 70 m from the top of the bedrock due to the strong physical weathering and chemical dissolution.

Effective porosity in Wellblock Dp17 is under 7.2% by helium measurements, and the maximum permeability is less than1.0 mD. Though shallow metamorphic limestone occur in conjunction with thin layer slate, the porosity calculated by acoustic curves change more evenly with the increase of burial depth, and the maximum porosity is less than 10.3% within the drilled depth (Fig. 6c). The reason for the uniformity of porosity development should be the uniform development of shorter fractures and secondary dissolution pores in limestone.

## Discussion

### Response of fractures to tectogenesis

Fractures of tectonic origin are one of the fundamental components of effective porosity^22,23^. In the meanwhile, high porosities and permeabilities are likely to associate with tectonically induced fractures because such fractures can significantly increase the permeability^24^. Altun Mountains experienced a strong left-lateral strike-slip movement, accompanied by a strong thrust-squeezing during the Cenozoic, causing a modern uplift in the Dongping area in the eastern section along with the entire movements^25,26^. Though multi-stage tectonic movements control the development of faults and tectonic fractures, the distance from the activity belt determines the extent and density.

From Wellblock Dp 3 to Wellblock Dp 17, the high uplift gradually transformed into the low slope area towards the center of basin, and the lithology changed as well, which resulted in different degrees of fracture development in the bedrock in different wellblocks under the action of the regional stress field. Wellblock Dp3 is located in a high fault-uplift zone with more steeply dipping and intensively denuded strata, where the basement rocks bear giant tectonic stress, enabling the tectonic fractures to modify and strengthen as well as form long and wide high angle fractures that are superior conditions for future basement reservoir improvement and to lay the foundation for the criss-crossed pore-fracture systems in the granite^27^. Wellblock Dp 1 is located in the slope zone, with the main lithology being granitic gneiss. The effect of tectonic movements on fractures was not as strong as that in Wellblock Dp3, leading to high angle fractures reduce and low angle fractures increase. Partial dark mineral orientation was formed under high temperature and stress conditions, whichpromoted the fine dark-colored crack strips appear in groups and are stable in appearance. Meanwhile, the development of fractures can give rise to secondary porosity around them because flow mainly occurs in fractures, which creates more effective reservoir space in the basement rocks^23^. Wellblock Dp17 is closer to the center of the basin in comparison with the other two wellblocks; thus the tectonic movement contributes less to the formation of high angle fractures, mainly low angle cleavage fractures and dissolution fractures instead.

### Response of reservoir space to lithology

Lithology is a complex factor particularly in the diverse basement rock composition in different wellblocks, which causes abrupt lateral variations in matrix properties^28^. Quartz, feldspar, calcite and other brittle minerals directly facilitate the development of fractures in basement reservoirs, and chemically unstable minerals such as feldspar, calcite, mica and hornblende are easily dissolved to form dissolved pores in the study area. Granite in Wellblock Dp 3 and granitic gneiss in Wellblock Dp 1 are rich in quartz and feldspar, brittle rock mass that enhanced high fractures density and formed the majority of the effective pore space. Affected by tectonic movement, the abundant calcite developed in Wellblock Dp 17 generagtes cleavage easily. Soluble mineral such as feldspar, calcite, and mica around fractures was easily dissolved when the fluid flowed along the fractures channel.

The reservoir space also depends on the texture and structure of the basement rocks^29^. Block rocks and uniform rock are not prone to fractures or becoming inner curtains^30^, such as the blocky granite in Wellblock Dp 3. However, granular and strip-shaped heterogeneous rocks are usually prone to fractures and likely to becom excellent reservoirs, such as the granitic gneiss and calcite in Wellblock Dp 1and Wellblock Dp 17, respectively. The "dominant lithology" of the pore space in basement strata based on mineral content or rock structure is constructed, which is mainly developed in Wellblock Dp 1 and Wellblock Dp 17. Light and dark minerals in granitic gneiss in Wellblock Dp 1 are layered to form gneissic structure with “dominant lithology”, building reticulated fracture systems which are in favor of hydrocarbon accumulation, with high-angle tectonic fractures, low-angle cleavage and dissolution fractures,. The limestone in Wellblock Dp 17 is also featured by a “dominant lithology”, and dense short fractures develop here. On the whole, the difference in the fracture development in different lithologies led to the uneven distribution of fracture zones in the basement strata.

### Reservoir space response to near-surface processes

**Figure 7:**
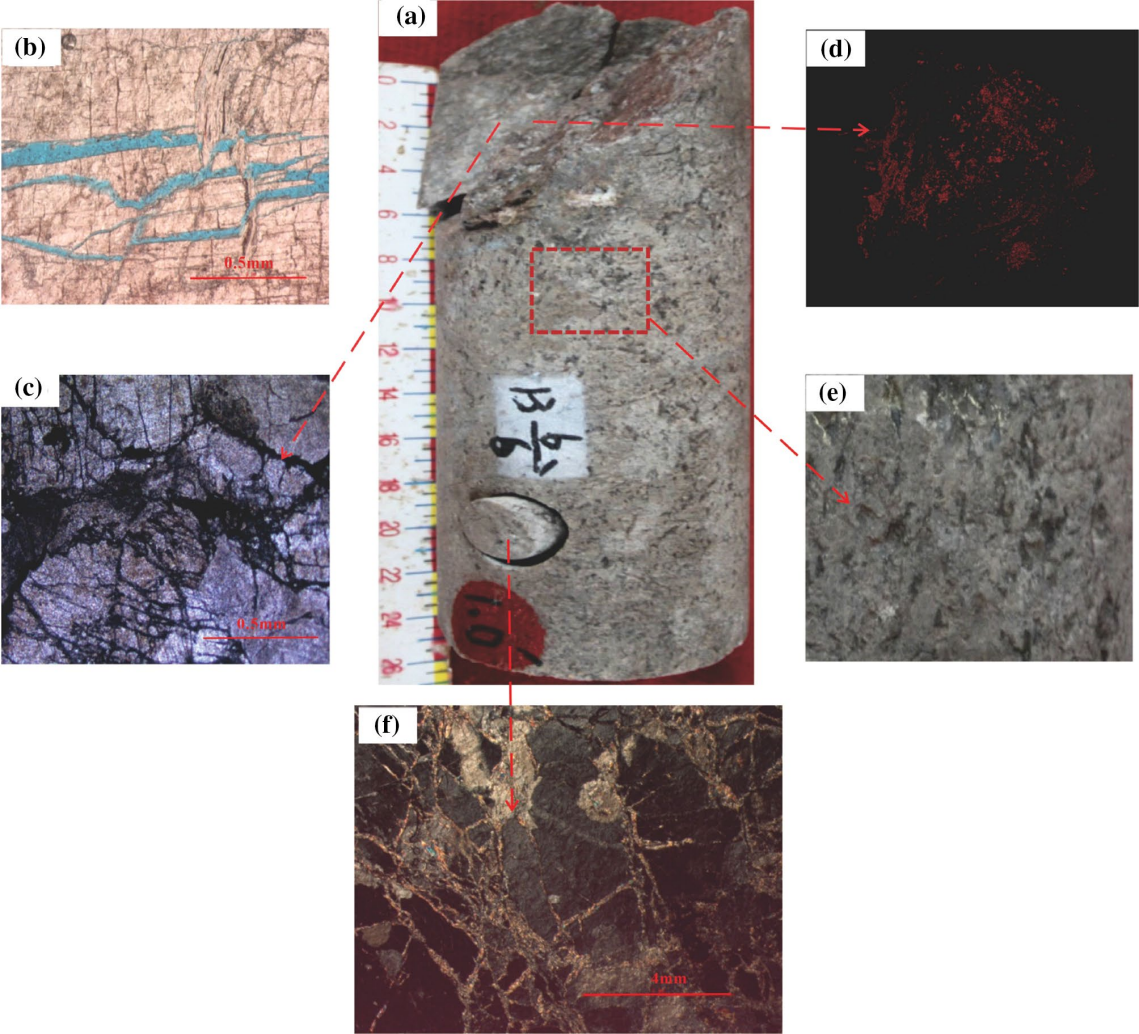
Focus on the fracture types and pores developed in the core of granitic gneiss. (**a**) Fractures and pores developed in granitic gneiss, which is 11.72.m from the top of the basement rocks in Well P1H-2–3 of Wellblock Dp1; (**b**) dissolved fractures developed along layer; (**c**) criss-crossed fractures; (**d**) dissolved pores measured by computerized tomography(CT) test; (**e**) dissolved pores on the surface of the basement rock; (**f**) dissolved fractures developed and some of them was filled but cemented.

Undergoing crushing and erosion through physical weathering and chemical solution especially in the structural fracture zone, the long-term outcropped rocks are poor in force resistance^31^. Basement rocks lying on the south-dipping slope in the Dongping area have been undergoing weathering, erosion, leaching, and solution for so long a time that solution fractures, joint fissures and cavermous solution pores have all been increasing greatly, so the accumulation of hydrocarbon is facilitated^13,15^. It is believed that the solution fracture is an important type of fracture that develop in the weathered-leaching zone by core and FMI observation in the study area (Figs. 4, 7). The upper part of the blocky granite was much broken due to a large number of joint fissures caused by strong weathering on the uplift, and a disorderly seepage system was formed as the flowing fluid underwent random movement in the bulk basement rocks (Fig. 3b). The layers of granitic gneiss developed on the slope that underwent leaching and weathering were also likely to form fractures and dissolved process (Fig. 7a,b). When fluid flew into opened fractures, unstable dark-coloured minerals along the tectonic fractures were easily dissolved and formed dissolution pores and vugs (Fig. 7c,d). These dissolved fractures and pores mainly developed along the layers in the plane, but the density of dissolved fractures and dissolved pores changed with the depth for filling happening in the basement rocks across the whole Dongping area (Fig. 7e,f). The Qaidam Basin is a saline lake environment during the Tertiary and the fractured-porous basement reservoirs are subjected to seepage and leaching of salt water. Calcite, gypsum and anhydrite gradually permeated the basement rocks and cemented and filled in the fractured-porous system. Ma^12^ found that fillings occurred in 0–18 m below the top of the basement rocks and it was in a gradual dropping trend from top down. As a result, physical weathering and chemical solution were likely to improve the physical properties of basement reservoir, while cementation and filling directly affected the seepage capability of the fractured-porous system in the basement rocks and the seepage capability gradually weakened with depth in the study area. Near-surface processes affected deeper basement rocks less. However, the physical properties of basement reservoirs in deeper locations mainly depended on the development of tectonic fractures, and it might be improved by the fluid with organic acid from hydrocarbon source^15^, just like metamorphic limestone developed in Wellblock Dp 17.

On the whole, one dominate feature is that the reservoir space of the basement shows huge spacial variation.. The degree of breaking and then weakening with the weathering and leaching gradually decreases towards the slope and low-lying area as the upper strata thickened^32^. Meanwhile, though some minerals were easily dissolved, causing fractures and pores in rocks, such as feldspar and mica, the secondary reservoir space induced by near-surface processes gradually reduced in vertical (Fig. 5). Koning^33^ found that the weathering of granites, especially under humid tropical conditions, can result in very porous secondary pores penetrating 100–200 m into the granite. However, considering the strong improvements of near-surface processes to the basement reservoirs and the decreasing trend of porosity along with the increasing depth in the basement rock reservoirs in Wellblock Dp 3 (Fig. 5), the depth limits of the high quality reservoir space should be about 200 m from the top of the basement rocks in Wellbolck Dp 3, while with the near-surface processes and a variety of porosities with the lithological characteristics combined, the depth limits of the high quality reservoir space should be about 100 m from the top of basement rocks in Wellbolck Dp 1 and 200 m from the top of the basement rocks in Wellbolck Dp 17.

### Gas distribution in different wellblocks

Though older basement rocks are dense and hard, long-term weathering and leaching can improve the properties of metamorphic and igneous rock reservoirs, increase the reservoir space, thus promoting oil and gas accumulation. However, The various development degrees of fractures and pores among these well areas exert powerful influence on the enrichment of natural gas.

**Figure 8:**
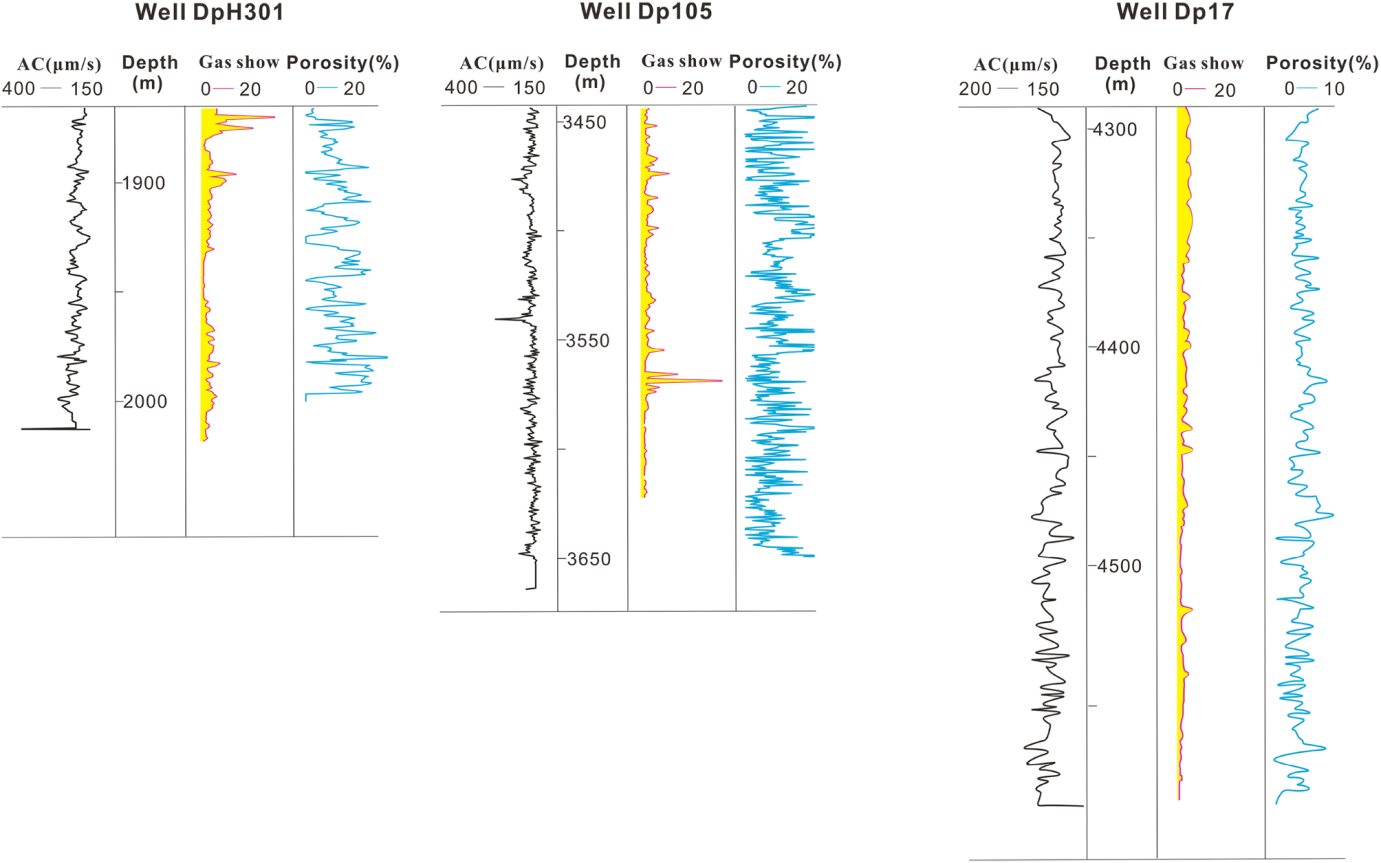
The correlation between gas content and porosity of typical wells in the three wellblocks.

Continuous gas layers in typical wells were found above the 200 m depth beneath the granite in Wellblock Dp 3 (Fig. 8). A section of 1878–2018 m, 12 m from the top of the basement rock, was tested in horizontal Well DpH301 by fracturing before high-yield industrial gas was obtained, with daily production of 12.1 × 10^4^ m^3^ through a 6 mm-diameter nozzle, whereas a section of 1858–2507 m, 127 m from the top of the basement rock, tested in horizontal Well Dp3H-6–2 by fracturing and daily production of 4.7 × 10^4^ m^3^ through a 6 mm-diameter nozzle was obtained. In Wellblock Dp 1, gas layers in typical wells were found under the 100 m depth beneath the granite (Fig. 8). A section of 3159–3182 m, 2 m from the top of the basement rock, was tested in Well Dp 1 by fracturing and high-yield industrial gas was also obtained, with daily production of 11.2 × 10^4^ m^3^ through a 6 mm-diameter nozzle. In comparison, a section of 3550–3.570 m, 114 m from the top of the basement rock, tested in well Dp 105 by fracturing and daily production of 2.2 × 10^4^ m^3^ through a 6 mm-diameter nozzle was obtained. In Wellblock Dp 17, gas layers in typical wells were found under the 200 m depth beneath the granite (Fig. 8). A section of 4338–4351 m, 39 m from the top of the basement rock, was tested in well Dp 17 by fracturing and high-yield industrial gas was obtained, with daily production of 5.14 × 10^4^ m^3^ through a 5 mm-diameter nozzle, while a section of 4778–4788 m, 401 m from the top of the basement rock tested in Well Dp 171 by fracturing and daily production of 5.76 m^3^ through a 4 mm-diameter nozzle was obtained. Analysis of the distribution mentioned above suggests that most of the productive wells are located in the weathering-leaching zones. The farther away from the top of the basement rock, the smaller the calculated porosity is. The dissolution fractures and dissolution porosity at the top of the basement rocks in all three wellblocks show a higher proportion, and the trial production is relatively high. In addition, the dissolution fractures and dissolution porosity show a gradually decreasing trend with the increasing of depth, the trial production is relatively low. The above description indicates that the differential gas content was determined by physical properties in the vertical direction. Due to the development of a large number of interactive micropores, dissolution pores and dissolution fractures, stratiform-like gas reservoir characteristics are displayed in weathering-leaching zones in the upper basement rocks in Dongping field^12^.

### Implication for future exploration

Although the basement reservoir in the Dongping field in Qaidam Basin has been explored successfully and some wells has been obtaining high yield, the distribution of gas accumulation in different wellblocks in the study area are highly differential, and they are also different from other large basement reservoirs in the world, such as the famous massive basement gas reservoirs in Cuu Long Basin, Liaohe Basin, and etc^33,34^.

The tectonic location, reservoir characteristic and sealing condition are considered as the the fundemental factors for gas accumulation in basement reservoirs^35^. Wellblock Dp3, located in the high fault-uplift zone, provides advantageous conditions for hydrocarbon migration and accumulation. Stronger weathering-leaching process builds higher porosity in granite. However, researchs on the capping conditions of the strata overlying the bedrock show that the overlying strata of basement rock is proximal coarse clastic sediments of a set of Paleogene alluvial fan facies belts as the research area is located in the piedmont zone of Altun Mountain^12^. The caprock could not successfully prevent the upward migration of natural gas as it did not have superior sealing conditions. Finally, natural gas could not be effectively sealed in the basement rock reservoir, from which it migrated upward until meeting a tight mudstone caprock and accumulating in the Paleogene strata in Wellblock Dp3^15^.

“Up-slope” theory of oil and gas migration in basement reservoirs are generally accepted. Wellblock Dp1, located on the slope in the eastern part of the nose structure, offers advantageous conditions for hydrocarbon migration and accumulation. A large number of brittle minerals and easily dissolved minerals form dense low-angle fractures and highly-developed bedding-parallel dissolution pores,the guarantee of high and stable yield. However, as the effective depth of reservoir modified by weathering-leaching is about 100 m and dissolution pores developed layer by layer, drilling horizontal wells within 100 m is important to the natural gas exploration in the slope area is proposed according to the above research.

In Wellblock Dp17 area, although the reservoir features good lithology which is conducive to the development of pores and fractures, it is located in the low-depression area and has no accumulation advantage due to the upward movement of gas along the fault and fractures. The gas reservoirs in deeper trap might deserve attention for future exploration.

## Conclusions

Tectonism, physical weathering, chemical leaching and different lithology are important factors that affect the development of reservoir space in the Dongping area. Tectonic fractures are formed by multi-stage tectonic movements throughout the basement rocks. In the shallow crust in Wellblock Dp 3, there are a many of highly permeable fractures that contribute the most to the store space in the basement reservoir, including high-angle tectonic fractures and a large quantity of boundary-solution fractures developing along with tectonic fractures. In the shallow crust in Wellblock Dp 1 and Wellblock Dp 17, there are also a many of highly permeable fractures that contribute the most to the store space in the basement reservoir including a lot of low-angle solution fractures developing along with soluble mineral layer. Weathering and leaching also exerts positiveeffects on a large number of solution pores and caves developing in the shallow crust. However, near-surface process weakens gradually with the increase of the burial depth, and different palaeogeomorphology and lithologies develope reservoirs with different physical properties. Effected by the solution process and "dominant lithology", the physical properties of the reservoirs varied remarkably with the different depths in different wellblocks. Three peak areas appeared in the porosity in Wellblock Dp 3 and Wellblock Dp 1, respectively. The porosity changed more evenly with the increasing of the burial depth in Wellblock Dp 17. The change of porosity in the basement rocks led to high productivity layers above the 200 m, 100 m and 200 m depths from the top of basement rocks in Wellblock Dp 3, Wellblock Dp 1 and Wellblock Dp 17, respectively. Therefore, the more favorable exploration area should be the uplift and slope area in the depression area. The discovery of the differential accumulation suggests that significant exploration potential remains in the basin in front of the Altun Mountain, and the new play concepts are crucial for future exploration in this area.
